# Low-dose CT examination for rib fracture evaluation: A pilot study

**DOI:** 10.1097/MD.0000000000011624

**Published:** 2018-07-27

**Authors:** Liang Jin, Xiaojun Ge, Fang Lu, Yingli Sun, Cheng Li, Pan Gao, Feng Gao, Ming Li

**Affiliations:** Department of Radiology, Huadong Hospital, Affiliated to Fudan University, Shanghai, China.

**Keywords:** noise, radiation, radiography, rib fracture, thoracic, tomography, X-ray computed

## Abstract

The aim of this study was to assess the applicability of low-dose thoracic computed tomography (CT) in the diagnosis of rib fractures.

A total of 37 trauma patients were selected for CT scanning using a noise index (NI) model. Each patient was scanned at both NI = 11 and NI = 26, while the other scanning parameters were kept the same. The scanning dose length product (DLP) and effective dose (ED) were recorded after each examination. Two radiologists diagnosed the rib fractures by degree (I, II, III, and IV) using Bone Reading software and axial images. Image quality was scored by 2 experienced radiologists using a 5-point scale. The numbers and degrees of rib fractures for different NIs were recorded and tested using the Chi-squared test. The interobserver differences were determined by kappa statistics.

The CTDIvols and EDs for NI = 11 and NI = 26 were 9.82 ± 4.78, 5.75 ± 2.75, and 2.14 ± 1.19 and 1.24 ± 0.73, respectively; the latter was decreased by 78.2% and 78.4% relative to the former. Low-dose thoracic CT was feasible for the auxiliary diagnosis of rib fractures using Bone Reading software (*P* > .05). There was perfect interobserver concordance in terms of diagnostic acceptability (kappa = 0.931, 0.905).

The use of an appropriate low-dose CT scanning technique is satisfactory for the assessment and diagnosis of rib fractures.

## 1 Introduction

With ongoing developments in society, the incidence of accidents and resulting chest trauma have also increased. One study found that from 2000 to 2005, the number of chest computed tomography (CT) examinations of emergency room patients increased by 226%, the number of patients with chest trauma increased by 13%,^[1]^ and chest trauma accounted for 10% to 15% of all traumatic injuries.^[2]^ Among chest trauma patients, 10% of the patients had rib fractures.^[3]^ Rib fractures are often accompanied by severe pain and complications such as hemothorax, pneumothorax, and spleen ruptures.^[4,5]^

**Figure 1:**
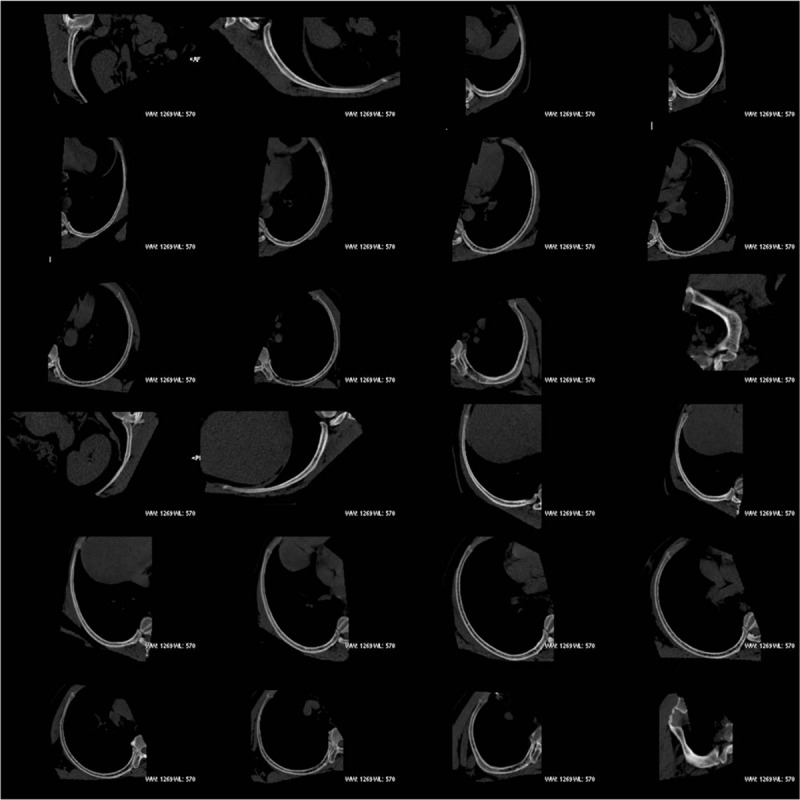
CPR images of 24 ribs (normally, 1 individual has 24 ribs).

A chest radiograph will show serious complications such as effusion, hemothorax, or pneumothorax; another frequent occurrence is bruised or fractured ribs, which are often treated supportively with analgesia. However, for many patients with major acute trauma (e.g., car crash, accident), the patient may not be capable to cooperate for chest radiograph. In addition, chest radiograph does not provide high sensitivity for small fractures and cannot clearly display lesions in the chest cavity or chest wall.^[6]^ If a diagnosis cannot be confirmed promptly, the subsequent treatments may be negatively influenced. However, the number of rib fractures is also an important indicator in forensic examination for degree of disability. Sometimes, defining rib fractures is very difficult for radiologists.^[7]^ Since 2002 in our hospital, we have used conventional curve planar reformation (CPR) (Fig. 1) in suspected rib fractures for forensic reasons. The goal of this approach was to decrease the workload of radiologists for the diagnosis of rib fractures while maintaining diagnostic accuracy. Since its emergence, Bone Reading software has been considered much more effective and more accurate than conventional diagnostic procedures.^[8]^

Therefore, traditional chest CT performs better in revealing occult fractures and fracture-related complications; it is often the primary choice of examination for chest trauma patients.^[9–11]^ However, the radiation dose of traditional chest CT is typically multiple times higher than that of regular chest imaging. The higher dose impedes the promotion of applying CT in chest trauma diagnosis.

In recent years, low-dose CT use became a trend worldwide. The number of studies reporting clinical applications of low-dose chest CT has increased; these reports include studies using low-dose chest CT in the screening, diagnosis, and follow-up for small nodules in lungs.^[12–18]^ Meanwhile, as time goes on, more and more image processing technologies come into view.^[19–21]^

The focus of our study was the implementation of a low-dose scanning technique for imaging of rib fracture patients. We compared the differences between normal-dose and low-dose imaging in displaying rib fractures. Furthermore, we investigated the feasibility and the practicality of low-dose chest CT examination for rib fracture patients.

## 2 Materials and methods

Thirty-seven patients underwent thoracic or chest-abdominal CT scans in our hospital from 2012 to 2016, and the patients with complete thoracic CT data were selected (n = 37; 22 male and 15 female; age range 34–72 years; average age 51.21 ± 8.50 years). The patients’ heights, weights, and body mass indexes (BMIs) were recorded before CT examination, and informed consent forms were signed.

### 2.1 Scan method and technique details

A Gem CT scanner (Discovery HD750; GE Healthcare, Waukesha, WI) was employed. Patients were asked to inhale and hold their breath during the scan. Scans ranged from the thoracic opening to the lower edge of the 12th rib. An automatic milliamp (SmartmA; GE Healthcare, Waukesha, WI) exposure scanning technique was applied. The tube voltage was set at 120 kV, the pitch was 0.984, the anode rotation time was 0.5 s, and the scan thickness was 5 mm in 5-mm intervals. A bone reconstruction algorithm was employed (window width 2500 HU, window level 300 HU). Axial reconstruction was performed; the layer thickness was 0.625 mm with 0.625-mm intervals.

This study was approved by the Ethics Committee of our hospital. Patients were examined using regular-dose CT [noise index (NI) = 11] as group A and low-dose CT (NI = 26) as group B. Under the NI mode, the range of auto-adjustment was 10 to 650 mA.

### 2.2 Image post-treatment

The scans and reconstructed images of all the patients were sent to Syngo (Siemens, Erlangen, Germany) via workstation through a Picture Archiving and Communication System (PACS). The built-in Bone Reading software generated the rib-unfolding visual effect based on the thin-section CT data.

**Figure 2:**
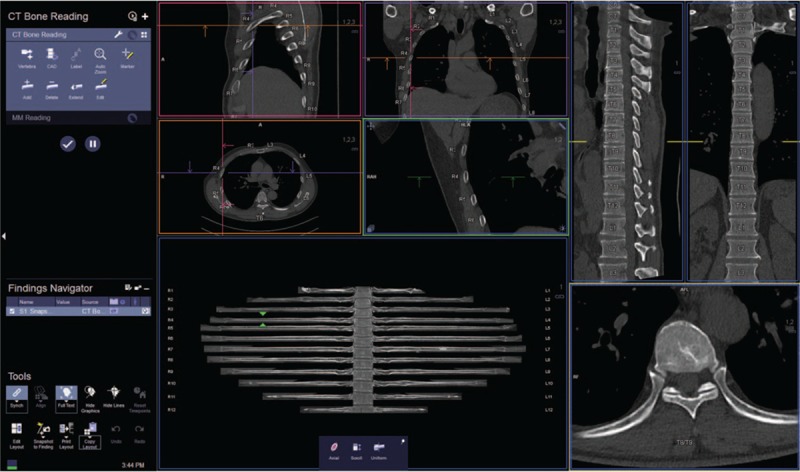
Bone Reading images.

The working principle of the software is the confirmation of the centerlines of all rib bones by pairing with the built-in models. Using the CPR reconstruction technique along the centerline, the unfolded thoracic image was obtained. The unfolded image of each rib was linked to the spinal cord, and the left and right sides and the codes were illustrated. The entire post-treatment only required 1 minute. The transverse, coronal, and sagittal plane multiplanar reconstruction (MPR) images could be obtained simultaneously^[10]^ (Fig. 2).

**Figure 3:**
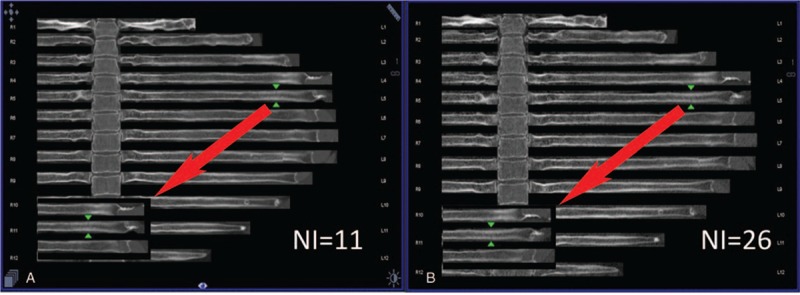
(A, B) Images taken with NI = 11 and NI = 26. The fresh fracture on the left 5th rib was a type I fracture. The green arrow is pointing at the fracture. The white arrow indicates the insert containing the enlarged image of the fracture.

**Figure 4:**
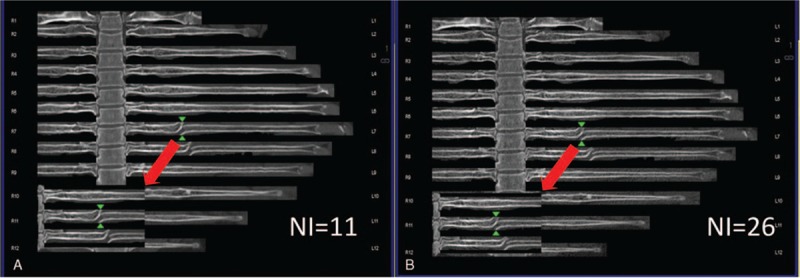
(A, B) Images taken with NI = 11 and NI = 26. The left 7th and 8th ribs had apparent fractures (type II) with dislocations.

**Figure 5:**
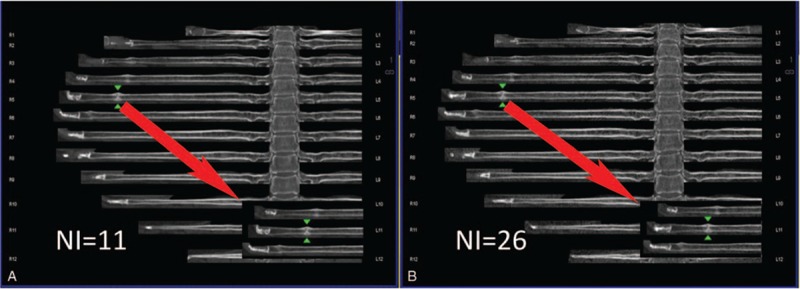
(A, B) Images taken with NI = 11 and NI = 26. The right 3rd, 4th, 5th, and 6th ribs were fractured (type III) with calluses formed.

**Figure 6:**
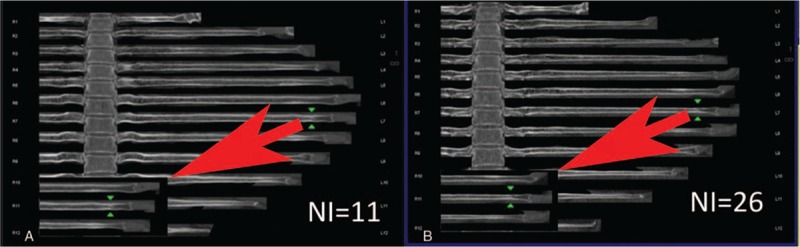
(A, B) Images taken with NI = 11 and NI = 26. The left 7th and 9th ribs were type IV fractures and were in the healing phase.

Research coordinators then evaluated the accuracy of the rib centerline and the integrity of the unfolded images. If the rib centerline was incomplete or inaccurate or if the thoracic images did not unfold completely, then a manual correction was performed by the research coordinators. The software also supports manual deletion, addition, and editing functions. In clinical work, the complete unfolded rib images are evaluated and corrected by radiology technicians (Figs. 3–6).

Names were removed from the image data of all included patients, and the images were then saved in DICOM format. The raw transverse plane image and the posttreated image were viewable by patient codes.

### 2.3 Image quality evaluation

Two experienced radiology physicians with more than 15 years of experience in image diagnosis performed double-blind evaluations on the raw images obtained using different NIs and on the post-treated images using Bone Reading software to diagnose rib fractures. The 5-point scoring system employed by Kalra^[14]^ was referenced for image evaluations. The acceptance rate of image diagnosis was evaluated using the following scale: 1 - insufficiency, where the diagnosis requirements were completely not met; 2 - below standard, where the diagnosis requirements were not met; 3 - average performance, where the diagnosis requirements were met; 4 - good performance, where the diagnosis requirements were met; and 5 - outstanding performance, where the diagnosis requirements were met. Images with scores ≥3 were considered applicable in clinical diagnosis. Subjective noise scoring was as follows: 1 - noisy image; 2 - higher than acceptable noise level; 3 - acceptable noise level; 4 - less than average noise level; and 5 - very minor noise level. An acceptable noise level was defined as an image that can satisfactorily reveal cortical bone, subtle fracture lesions, and vertebral trabecular bone and can show clear boundaries for nearby tissues.

On the basis of our experience in diagnosis, we classified the rib fractures into 4 types: type I (Fig. 3) - fresh fracture without dislocation; type II (Fig. 4) - fresh fracture with apparent dislocation; type III (Fig. 5) - old fracture with a callus; and type IV (Fig. 6) - old fracture with distortion after healing or an old fracture with doubtful completeness of healing.

### 2.4 Radiation dose

The auto-generated dose length product (DLP) was recorded from the equipment. The DLP was then multiplied by K (a conversion coefficient) to obtain the effective dose (ED = DLP∗K) for each patient. During the thoracic-abdominal scan for rib trauma fracture, a K of 0.014 was used.^[22]^

### 2.5 Statistical analysis

In this study, IBM SPSS Statistics 22 (IBM Corp., Chicago, IL) was employed for the statistical analysis. A Chi-squared test was applied to analyze the results of examinations based on thin section transverse cross-sections using different NIs and based on post-treated images. The consistency of the evaluations given by the 2 physicians was determined by kappa analysis. The kappa values were defined as follows: <0.20, almost inconsistent; 0.21 to 0.40, slightly consistent; 0.41 to 0.60, moderately consistent; 0.61 to 0.80, highly consistent; and 0.81 to 1.00, almost perfectly consistent. Differences with *P* values <.05 indicate statistical significance.

## 3 Results

The average CTDIvols were 9.82 ± 4.78 and 2.14 ± 1.19, while EDs were 5.75 ± 2.75 and 1.24 ± 0.73 for NI = 11 and 26, respectively. The CTDIvol and DLP of the latter was decreased by 78.2% and 78.4% compared with that of the former (Table 1). Among the 37 patients, 1 case was eliminated due to failed post-treatment by the Bone Reading software. Another case had a congenital bilateral absence of the 12th ribs. Therefore, in this study, thin-section axial images combined with post-treatment scans using Bone Reading software were applied to evaluate rib fractures in 862 rib images from 36 patients. A total of 187 and 184 ribs were determined to be fractured at NI = 11 and 26, respectively. When NI = 11 and NI = 26, the number of ribs diagnosed to be fractured was 187 and 184, respectively. When NI = 11, there were 23 type I fractures, 9 type II fractures, 108 type III fractures, and 47 type IV fractures, while when NI = 26, 23 type I, 8 type II, 108 type III, 45 type IV. The differences were statistically insignificant (Table 2).

**Table 1:**
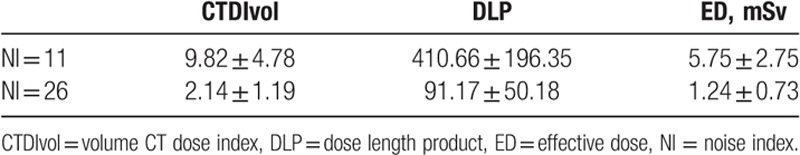
Comparison of radiation doses in sequential scans with different NIs.

**Table 2:**
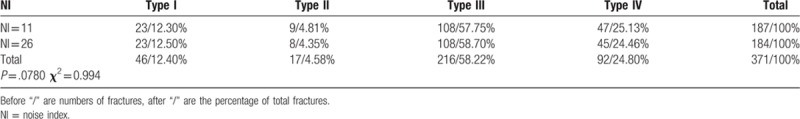
Comparison of fracture detection results between different NI scans.

The acceptance rates for axial image diagnosis with scan conditions applied to groups A and B and the subjective noise scoring data from the 2 physicians are provided in Table 3. When NI = 11, the averaged image diagnosis acceptance rate and subjective score were 4.94 ± 0.22 and 4.78 ± 0.42, respectively; when NI = 26, the rate and the score were 2.85 ± 0.36 and 2.62 ± 0.48, respectively. These data indicate that the higher the NI is, the lower the radiation dose, acceptance rate, and subjective score. However, there was no statistically significant difference in diagnosis. The 2 physicians performed a comprehensive analysis of the thin-section axial images. The provided acceptance rate and subjective noise score had almost perfect consistency between the 2 physicians (the kappa values were 0.931 and 0.905, respectively).

**Table 3:**
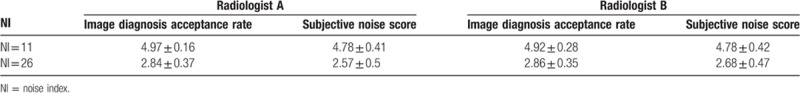
Image diagnosis acceptance rate and subjective noise score.

## 4 Discussion

With ongoing developments in CT technology, CT examination has become more applicable in many fields, which has resulted in increases in the frequency of clinical exams and radiation doses. The amount of public attention paid to radiation has also gradually increased. Therefore, personalized scans should be applied for every CT examinee. In other words, under the premise of satisfying the requirements of examination and diagnosis, the as low as reasonably achievable (ALARA) principle should be followed, which will significantly affect clinical practice.^[23]^

Chest x-ray imaging plays an important role in evaluating and managing blunt chest trauma. Due to its characteristics such as rapidness and high accuracy and its ability to provide additional information for diagnosis, multislice helical CT has become the most important imaging method.^[24–26]^

In this study, the tube voltage was automatically regulated via applying the default NI value given by the CT instrument, and the goal of lowering the scan dose was achieved. As the NI increases, the average tube current decreases, as does the radiation dose. Due to the decrease in the tube current, certain noise levels are generated in the images of the examined organs. In relative terms, for solid organs such as the liver, spleen, and kidney, the object contrast is lower due to their similar tissue densities; therefore, the images of these organs are more susceptible to the influence of noise. For the ribs and lungs, where the object contrast is higher, the influence from noise is lower; hence, low-dose thoracic CT is feasible. Currently, the manufacturer recommends NI = 10 to 11 as the default value for a regular thoracic CT scan. The 5-level evaluation of the application of different NI values performed by Kalra^[14]^ recommended NI = 12 to 15 for adult thoracic CT scans. In this study, NI = 11 was selected as the regular scan dose. With the lower limit of the tube current at 10 mA, NI = 26 was used for the low-dose scan. The results indicated that as NI increased, the radiation doses received by the patients greatly decreased.

With the gradual improvements in CT differentiation and ongoing developments in post-treatment techniques, physicians increasingly choose CT for the evaluation of trauma-related rib fractures. Specifically, in forensic examinations, CT has advantages in diagnosing the numbers and types of rib fractures. However, image diagnoses for rib bones are complicated by the number of ribs, individual differences in rib morphology, morphological changes after fracture, the occurrence of occult fracture, and other factors. With Bone Reading software capable of 3-dimensional reconstruction, the time for such a reconstruction of the rib bones can be greatly reduced when using regular-dose CT. This technology can save time and energy for radiology physicians and can provide a certain level of assistance to the physician in rib fracture diagnosis.^[10]^

This study, which was based on the observations made on raw axial images versus Bone Reading post-treated images, aimed to further investigate the value of low-dose chest CT in rib fracture diagnosis. The results of this study indicate that the consistency between the diagnoses by the 2 physicians reached 98.4% when observing images obtained using regular-dose CT (NI = 11) and low-dose CT (NI = 26); for NI = 11, the average image diagnosis acceptance rate and subjective score were 4.94 ± 0.22 and 4.78 ± 0.42, respectively, and for NI = 26, the values were 2.85 ± 0.36 and 2.62 ± 0.48, respectively. These results indicate that the image quality dropped slightly at NI = 26 compared with those at NI = 11. However, the decrease in image quality did not influence the diagnosis and evaluation of rib fractures. Therefore, the application of low-dose thoracic CT in rib fracture evaluation is feasible. Low-dose CT can effectively reduce the radiation dose received by the examinee, with an average ED as low as 1.24 ± 0.73 mSv.

The application of low-dose CT in rib fracture diagnosis has been reported by other researchers. There is a great potential benefit to use low-dose CT for the initial evaluation of blunt chest trauma because low-dose CT could maintain the same diagnostic image quality as regular-dose CT and provide a significant radiation dose reduction. The effective radiation dose of low-dose CT (average DLP = 1.52 mSv·mGy/cm) was significantly lower than that of regular dose CT (7.21 mSvmGy/cm).^[27]^ In our study, it was experimentally determined that the ED could be further decreased and that the image quality remained satisfactory for diagnosis and the low-dose protocol was more convincing as well.

However, in our study, some insufficiencies of low-dose thoracic CT were also identified. Images of 1 case could not be read using the Bone Reading software. Further investigation found that the difficulty in imaging reading could be attributed to the severe deformity of the near-spinal-cord ribs resulting from thoracic compression fractures. In images obtained using low-dose CT, the software could not recognize the images for acceptable rib unfolding and reconstruction. In addition, application of a low-dose scan in the diagnosis of a fresh fracture without dislocations could yield inconsistent results due to the unclear visualization of regions near the rib cartilage. For type IV old fractures with distortions after healing or old fractures with unconfirmed completeness of healing, there are even greater difficulties and a lack of confidence in providing an accurate diagnosis.

There are some limitations in this study. First, rib diagnosis requires sufficient experience. The acceptance of the application of low-dose thoracic CT in rib diagnosis could be different among physicians with different degrees of experience, especially for radiology physicians with only a few years of practice; further studies and investigations should be performed. Second, at present, researchers have found that thoracic CT images taken with even lower radiation doses with NIs greater than 26 could satisfy the requirements for diagnosis. Further studies and investigations should be performed to evaluate their applicability in rib fracture diagnosis.

## 5 Conclusion

Through our research, we proved that the use of an appropriate low-dose CT scanning technique is satisfactory for the assessment and diagnosis of rib fractures. This low-dose CT technique can be used for accurate diagnosis of rib fractures.

## Acknowledgements

This study was supported by Health Commission of Shanghai, Wise Information Technology-Major Program of Medical Imaging(2018ZHYL0103) and the Research Program of Shanghai Hospital Development Center (SHDC22015025).

## Author contributions

**Conceptualization:** Liang Jin.

**Data curation:** Liang Jin.

**Formal analysis:** Feng Gao.

**Investigation:** Xiaojun Ge, Cheng Li.

**Methodology:** Xiaojun Ge.

**Project administration:** Ming Li.

**Resources:** Ming Li.

**Software:** Fang Lu, Yingli Sun, Pan Gao.

**Writing – original draft:** Liang Jin.

**Writing – review & editing:** Ming Li.
